# Effect of a Solid-Hydrogen Environment on UV-Induced Hydrogen-Atom Transfer in Matrix-Isolated Heterocyclic Thione Compounds

**DOI:** 10.1021/acs.jpca.1c05538

**Published:** 2021-08-18

**Authors:** Hanna Rostkowska, Anna Luchowska, Leszek Lapinski, Maciej J. Nowak

**Affiliations:** Institute of Physics, Polish Academy of Sciences, Al. Lotników 32/46, 02-668 Warsaw, Poland

## Abstract

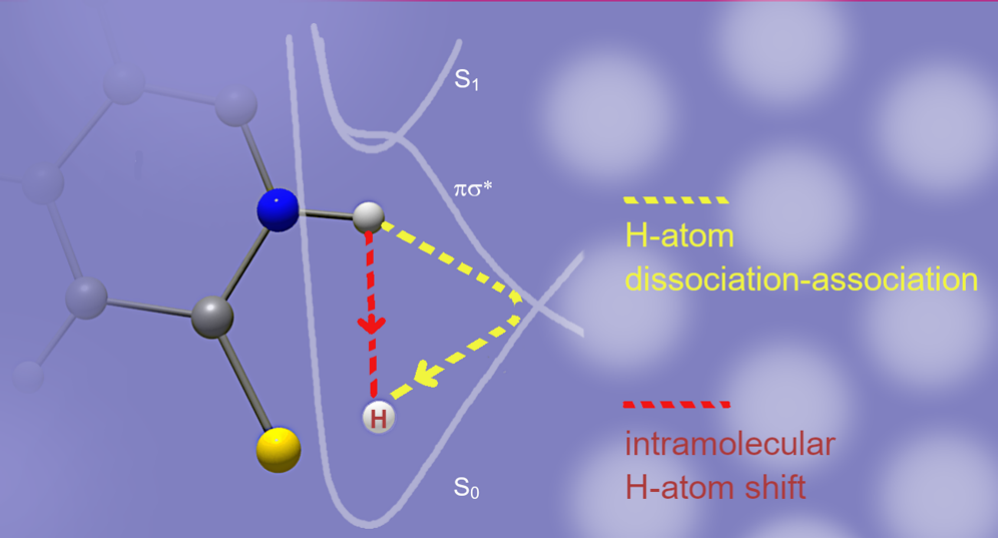

To shed more light on the mechanisms of UV-induced hydrogen-atom-transfer processes in heterocyclic molecules, phototautomeric thione → thiol reactions were investigated for thione compounds isolated in low-temperature Ar as well as in n-H_2_ (normal hydrogen) matrices. These studies concerned thione compounds with a five-membered heterocyclic ring and thione compounds with a six-membered heterocyclic ring. The experimental investigation of 2-thioimidazole and 3-thio-1,2,4-triazole (thione compounds with a five-membered heterocyclic ring) revealed that for the compounds isolated in solid n-H_2_ only trace amounts of thiol photoproducts were photogenerated; even though for the same compounds isolated in the solid Ar matrix, the thione → thiol photoconversion was nearly total. In contrast to that, for 3-thiopyridazine and 2-thioquinoline (thione compounds with a six-membered heterocyclic ring) isolated in solid n-H_2_, the UV-induced thione → thiol conversion occurred with the yield reaching 25–50% of the yield of the analogous process observed for the same species isolated in solid Ar. The obtained experimental results allow us to conclude that the dissociation–association mechanism nearly exclusively governs the phototransformation in thione heterocycles with high barriers for tautomerization (such as thione compounds with a five-membered ring), whereas the strictly intramolecular hydrogen-atom shift contributes to the mechanism of hydrogen-atom transfer in thione heterocycles with lower barriers (such as thione compounds with a six-membered ring).

## 1 Introduction

**Scheme 1:**
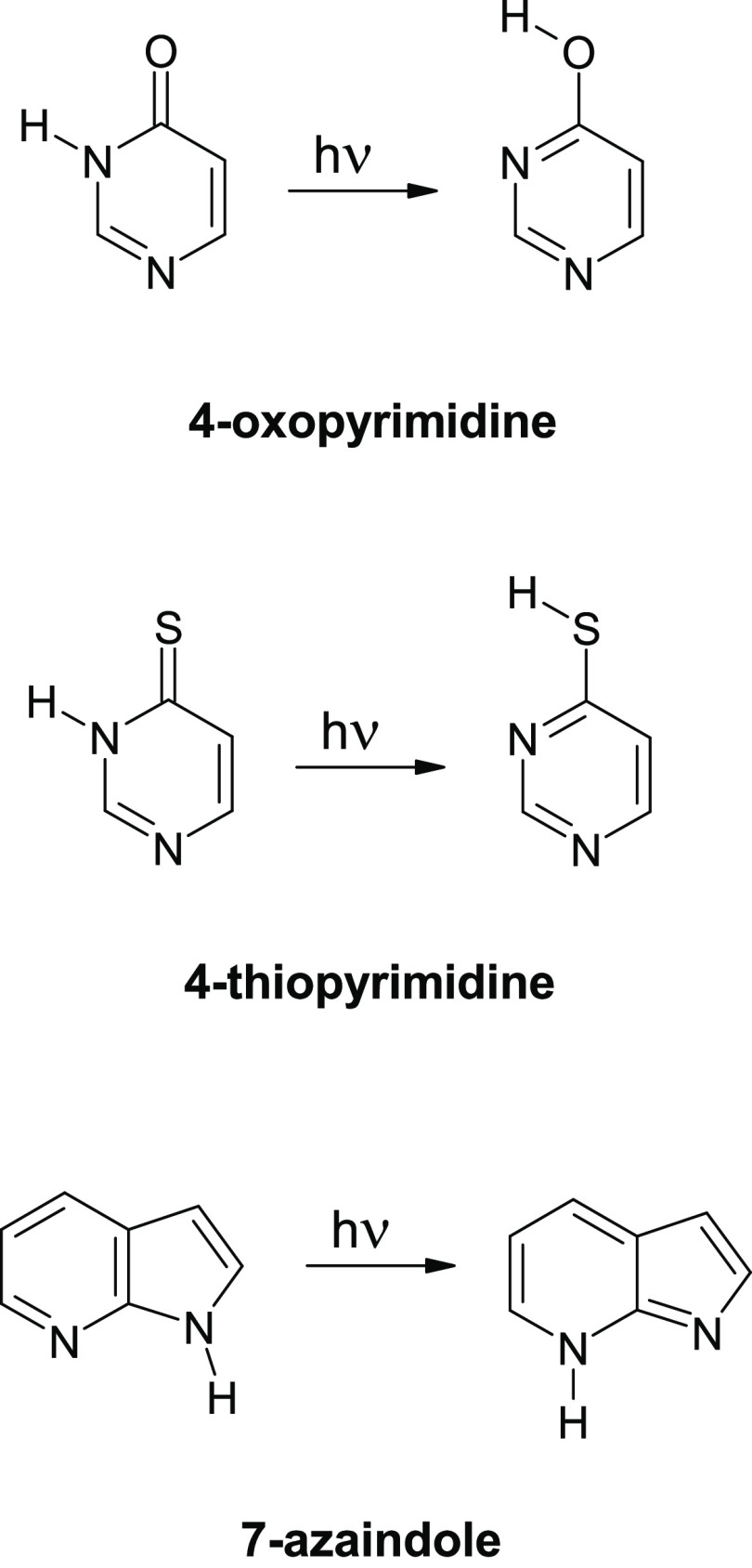
Examples of UV-Induced Hydrogen-Atom-Transfer Processes (Phototautomeric Transformations) Observed for Monomers of Heterocyclic Compounds Isolated in Low-Temperature Matrices

A distinct class of intramolecular H-atom-transfer processes, leading to the oxo → hydroxy, thione → thiol, or N(*i*)H → N(*j*)H change of tautomeric form (Scheme 1), was observed for a number of heterocyclic compounds isolated in low-temperature argon matrices and exposed to UV light. These photoisomerization processes occur for molecules without any intramolecular hydrogen bonds in their structure. Moreover, for the molecules undergoing this type of phototautomerism, the energy gap between the S_0_ and S_1_ electronic states of the reactant tautomer is smaller than the corresponding S_0_–S_1_ energy gap in the photoproduced tautomer. Hence, in these systems, the hydrogen-atom transfer may not proceed in the way typical of the excited-state intramolecular proton transfer (ESIPT) processes,^1−5^ that is, on the potential-energy surface of the excited S_1_ electronic state.

Phototautomerization processes belonging to this category were observed for the compounds isolated in the matrices of solid Ar (or other noble gases) as well as in the matrices of solid N_2_.^6−29^ It appears that the UV-induced oxo → hydroxy, thione → thiol, or N(*i*)H → N(*j*)H hydrogen-atom transfer leading to the change of tautomeric form (phototautomerism) is a typical pattern of the photochemical behavior of monomeric heterocyclic compounds isolated in low-temperature Ar, Xe, Ne, or N_2_ matrices. Such phototautomeric reactions may proceed either by a mechanism involving detachment of a hydrogen atom followed by its association to another heteroatom or by a purely intramolecular mechanism involving a shift of a hydrogen atom that occurs during dissipation of the excitation energy, when the molecule is in highly excited vibrational levels of the ground electronic state.

The very weak van der Waals interactions between isolated molecules and their matrix environment should have very little effect on the progress of a strictly intramolecular phototransformation. The strength of the van der Waals interactions between a molecule and its solid Ar environment is nearly the same as the strength of the interactions between the same molecule and its solid-hydrogen environment. This is demonstrated by almost identical spectral positions (indicating similar matrix shifts) of IR bands observed in the spectra of molecules isolated in Ar and in normal hydrogen (n-H_2_) matrices.^28,30^ Assuming that the UV-induced hydrogen-atom transfer (occurring in an Ar matrix) is a purely intramolecular process, it would be only natural to expect that the analogous UV-induced hydrogen-atom transfer should also occur for the same molecules but isolated in normal hydrogen (n-H_2_) matrices.

The results of the recent studies on phototransformations, occurring for 7-azaindole, 4-oxopyrimidine, and 6-hydroxy-4-oxopyrimidine monomers isolated in Ar and n-H_2_ matrices and exposed to UV light, contradict this supposition. For the monomers of 4-oxopyrimidine and 6-hydroxy-4-oxopyrimidine isolated in Ar matrices and excited with UV light, oxo tautomers were converted into hydroxy tautomers by hydrogen-atom transfer from the N–H group to the vicinal oxygen atom.^30,31^ These phototautomerizations were accompanied by minor photoisomerization processes leading to the formation of the nonplanar Dewar isomers and the open-ring ketenes. Contrary to what was expected, UV irradiation of the same molecules but isolated in n-H_2_ matrices resulted solely in the formation of the Dewar isomers and the open-ring ketenes; the oxo → hydroxy phototautomeric conversion was not detected. Also, for 7-azaindole trapped in solid Ar and excited with UV light, the N(1)H → N(7)H phototransformation was clearly observed, whereas for monomers of the same compound isolated in n-H_2_ matrices, such phototautomeric conversion did not occur.^28^ Similarly, for 3-thio-1,2,4-triazole^32^ isolated in solid n-H_2_, only a trace amount of the thiol form was photogenerated, though for the same compound isolated in an Ar matrix, exposure to UV light led to an efficient transformation of the dominating thione tautomer into the thiol photoproduct.

Hence, phototautomerization processes are observed to efficiently proceed for 7-azaindole, 4-oxopyrimidine, 6-hydroxy-4-oxopyrimidine, and 3-thio-1,2,4-triazole isolated in Ar matrices, but no (or nearly no) analogous processes occur for these compounds isolated in solid n-H_2_. Such a different photochemical behavior, drastically dependent on an Ar or n-H_2_ matrix-environment, suggests that the strictly intramolecular mechanism does not substantially contribute to the overall mechanism governing these photoinduced tautomerizations.

The striking difference between the properties of solid Ar (and other noble gases Ne or Xe) and solid H_2_ concerns the “softness” of the latter matrix material.^33,34^ Matrix cages in noble-gas matrices, such as solid Ar, are substantially more rigid. This should make a difference for a phototransformation involving detachment of an atom (or a small group of atoms) followed by its reassociation to the matrix-isolated molecule. Therefore, if a photoisomerization in molecules of a compound isolated in solid Ar proceeds very differently from the reactions in molecules of the same compounds isolated in solid hydrogen, then one can conclude that the phototransformation in question is not a strictly intramolecular process.

In an attempt to propose the mechanism of the phototautomerism observed for compounds (such as 4-oxopyrimidine) isolated in low-temperature Ar matrices, only one theoretical model (known as PIDA, photoinduced dissociation association) has been formulated so far.^35^ According to this model, the phototautomeric transformation should concern a detachment of the hydrogen atom on the potential energy surface of the repulsive ^1^πσ* excited state followed by reassociation of this hydrogen atom to the main frame of the matrix-isolated molecule. Within such a scheme, the matrix environment may affect the progress of the phototransformation.

Although the experimental results obtained hitherto for heterocyclic compounds seem to support the conclusion that in these matrix-isolated species phototautomeric conversions are governed by a mechanism involving dissociation and association involving the labile hydrogen atom (PIDA), it is not necessarily the only mechanism explaining the phototautomeric conversions observed for matrix-isolated species. To recognize other mechanisms of hydrogen-atom transfer, in the present project, we have undertaken a more systematic experimental study of a number of heterocyclic compounds isolated in Ar and n-H_2_ matrices and excited with UV light. As the object of investigation, we have chosen a series of heterocyclic compounds containing thiolactam group attached to the five-, or six-membered ring. We also tried to relate the experimentally observed photochemical behavior of the investigated compounds to their structure and the height of ground-state barriers for change of the tautomeric form.

## 2 Experimental Section

2-Thioimidazole, 2-thioquinoline, thioacetamide, and 2-thiobenzothiazole experimentally investigated in the present study were commercial products purchased from Sigma-Aldrich [2-thioimidazole (98%), 2-thioquinoline (97%), thioacetamide (>99%)] or from TCI-Europe [2-thiobenzothiazole (>98%)]. 3-Thiopyridazine was prepared by the action of P_2_S_5_ on 3-pyridazinone purchased from Aldrich. Prior to a matrix-isolation experiment, a solid sample of the investigated compound was placed in a miniature glass oven, located inside the vacuum chamber of a cryostat with a Sumitomo SRDK-408D2 closed-cycle cooler. To prepare a low-temperature matrix, the cryostat was evacuated and the solid sample of the studied compound was heated by a resistive wire wrapped around the glass oven. The vapor of the compound was deposited, together with large (>1000) excess of Ar or normal H_2_ (n-H_2_), onto a CsI substrate cooled to 3.5 K. Mid-IR spectra of the matrices were recorded in the 4000–500 cm^–1^ range, with the 0.5 cm^–1^ resolution, using a Thermo Nicolet iS50R FTIR spectrometer equipped with a KBr beam splitter and a DTGS detector with a KBr window. The spectra in the 700–300 cm^–1^ range were recorded with the same spectrometer but equipped with a “solid substrate” beam splitter and a DTGS detector with a polyethylene window. The matrix-isolated compounds were irradiated with UV light emitted by 6060 LG Innotek diodes. In this work, two light-emitting diodes (LEDs) were applied: one with λ_max_ = 278 nm and the other one with λ_max_ = 305 nm. The spectral width (full width at half-maximum) of the light emitted by these diodes was 15 nm and the optical power of the generated UV light was 100 mW. In some experiments, filtered UV light emitted by an HBO 200 high-pressure mercury lamp was also applied to irradiate matrix-isolated compounds. For the compounds studied in the current work, the irradiation time was 240–420 min (see Figures S1, S2, and S6 in the Supporting Information).

## 3 Computational Section

Geometries of the tautomeric forms of the compounds considered in the present work were optimized at the DFT(B3LYP)^36−38^ and at the MP2^39^ levels of theory. At geometries optimized with the DFT(B3LYP) method, harmonic vibrational frequencies and infrared intensities were computed at the same level.

Heights of the barriers for thione → thiol, oxo → hydroxy, or N(1)H → N(7)H tautomerizations in the ground-electronic-state were assessed at the MP2 level. In the initial forms of the considered compounds, the labile hydrogen atom is attached to a nitrogen atom. From this N–H moiety, the hydrogen atom is transferred to a nearby located sulfur, oxygen, or another nitrogen atom. Every barrier height was calculated as a difference between the energy of the transition state (maximum on the minimum-energy path between two tautomers in question) and the energy of the initial tautomer [thione, oxo, or N(1)H]. All of the theoretical computations were carried out with the Gaussian 09, revision D.01, program^40^ using the standard 6-311++G(2d,p) basis set.

## 4 Results

### 4.1 UV-Induced Hydrogen-Atom Transfer in 3-Thiopyridazine and 2-Thioquinoline: Thione Compounds with a Six-Membered Heterocyclic Ring

**Figure 1:**
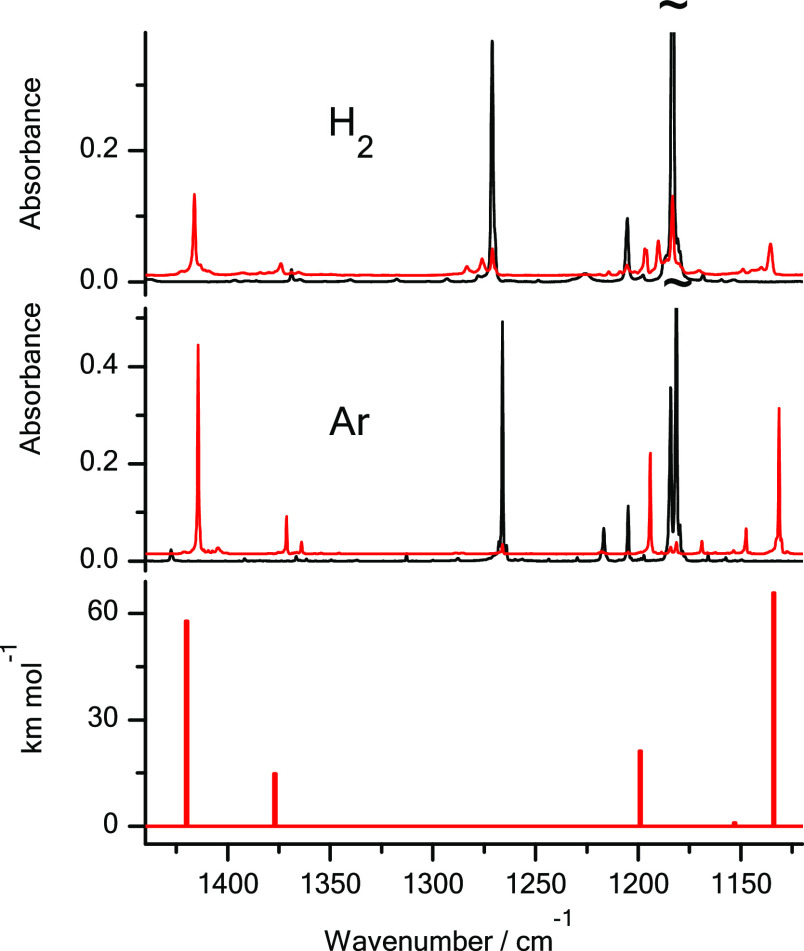
Fragments of the infrared spectra of 3-thiopyridazine monomers: (middle panel) isolated in an Ar matrix; (upper panel) isolated in an n-H_2_ matrix. Black traces represent the spectra recorded directly after deposition of the matrices at 3.5 K; red traces represent the spectra recorded after irradiation of the matrices with UV (λ = 305 nm) light. The experimental spectra are compared with the theoretical spectrum (bottom panel) calculated at the DFT(B3LYP)/6-311++G(2d,p) level for the thiol tautomer of the compound. The theoretical wavenumbers, calculated within the harmonic approximation, were scaled by 0.98.

**Figure 2:**
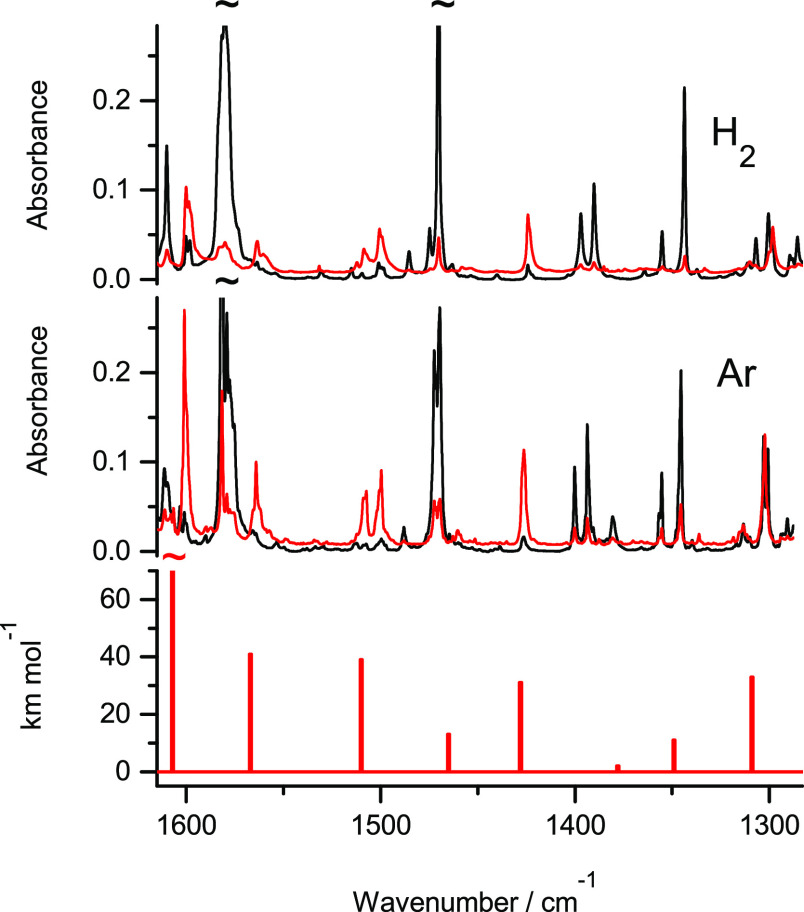
Fragments of the infrared spectra of 2-thioquinoline monomers: (middle panel) isolated in an Ar matrix; (upper panel) isolated in an n-H_2_ matrix. Black traces represent the spectra recorded directly after deposition of the matrices at 3.5 K; red traces represent the spectra recorded after irradiation of the matrices with UV (λ = 305 nm) light. The experimental spectra are compared with the theoretical spectrum (bottom panel) calculated at the DFT(B3LYP)/6-311++G(2d,p) level for the thiol tautomer of the compound. The theoretical wavenumbers, calculated within harmonic approximation, were scaled by 0.98.

**Scheme 2:**
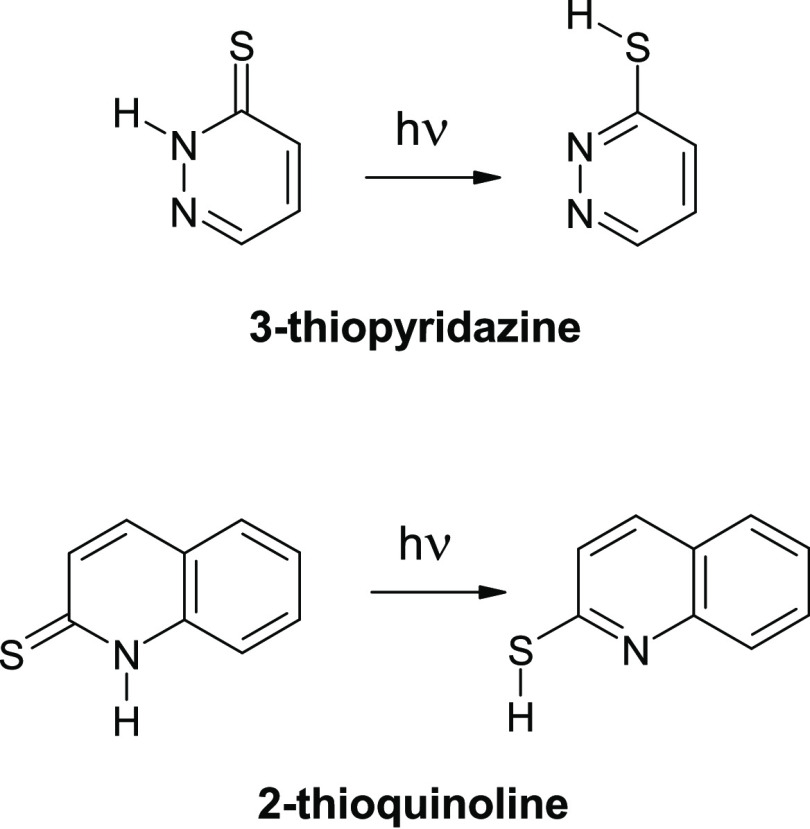
Thione → Thiol Phototautomeric Conversions Observed for Monomers of 3-Thiopyridazine and 2-Thioquinoline Isolated in Ar and n-H_2_ Matrices and Irradiated with UV (λ = 305 nm) Light

The UV-induced thione→thiol phototautomeric conversions were previously observed for 3-thiopyridazine and 2-thioquinoline isolated in Ar matrices.^14,19^ In the current study, the photochemical behavior has been examined for monomers of 3-thiopyridazine and 2-thioquinoline isolated in a solid n-H_2_ environment. Prior to any irradiation, the thione forms of 3-thiopyridazine and 2-thioquinoline were found to dominate in n-H_2_ matrices, analogously as the thione forms dominated for these compounds isolated in Ar matrices.^14,19^ UV excitation of 3-thiopyridazine and 2-thioquinoline isolated in n-H_2_ matrices led to photoproduction of the thiol forms of these compounds (Scheme 2 and Figures 1 and 2). In the IR spectra of the compounds isolated in n-H_2_ matrices and irradiated with UV (λ = 305 nm) light, the new bands appeared at the same (or very similar) spectral positions as the IR bands attributed to the thiol tautomers photogenerated from the thione forms of 3-thiopyridazine and 2-thioquinoline isolated in solid Ar (Figures 1 and 2).

Similarly, as it was the case for 2-thioquinoline isolated in solid Ar, some small population of the thiol form was found to exist also in the n-H_2_ matrix directly after its deposition. Hence, for 2-thioquinoline isolated in Ar and n-H_2_ matrices and irradiated with UV (λ = 305 nm) light, generation of the thiol tautomer manifested itself as intensity increase of very weak IR bands (Figure 2), which were already present in the spectra recorded before any irradiation. This is a further evidence that, similarly as in Ar matrices, the thiol tautomer of 2-thioquinoline was photogenerated also for the compound isolated in n-H_2_ matrices.

The results presented in Figures 1 and 2 demonstrate that a significant amount of thiol photoproducts was photogenerated for 3-thiopyridazine and 2-thioquinoline isolated in solid n-H_2_. However, these quantities are smaller than the amount of the thiol tautomers photoproduced for the compounds isolated in solid Ar. The yield of thione → thiol phototransformations, occurring for monomers of 3-thiopyridazine and 2-thioquinoline isolated in Ar matrices, is believed to be close to 100%.^14,19,20^ Close inspection of the spectra of 3-thiopyridazine and 2-thioquinoline isolated in solid n-H_2_ and irradiated with UV (λ = 305 nm) light (Figures S1 and S2 and the related comment presented in the Supporting Information) showed that the efficiency of UV-induced thione → thiol conversions in these compounds isolated in solid n-H_2_ can be assessed as 25–50% of the efficiency of the analogous thione → thiol photoconversion proceeding in Ar matrices. No quantum-yield assessment was attempted in the present work. The reason for that is the inherently poor reliability of the experimental procedures aimed at estimation of quantum yield of photoprocesses occurring in matrix-isolated molecules (see Note S1 in the Supporting Information).

This significant (25–50%) efficiency of the phototautomerization processes, observed for 3-thiopyridazine and 2-thioquinoline isolated in solid n-H_2,_ is quite surprising in comparison to the results obtained for other heterocycles (4-oxopyrimidine, 6-hydroxy-4-oxopyrimidine, 7-azaindole, and 3-thio-1,2,4-triazole)^28,30−32^ isolated in n-H_2_ matrices. For these latter compounds, UV excitation led to no (or nearly no) phototautomeric conversion. For 3-thiopyridazine, as well as for 2-thioquinoline, the heights of the barriers for the ground-electronic-state thione → thiol tautomeric conversion have been calculated, within the current work, at the MP2/6-311++G(2d,p) level. The obtained values (128 kJ mol^–1^ for 3-thiopyridazine and 127 kJ mol^–1^ for 2-thioquinoline) are noticeably lower than the analogous barrier heights computed for the N(7) → N(1) tautomeric hydrogen shift in 7-azaindole and for the oxo → hydroxy tautomerism in 4-oxopyrimidine (see Table 1).

**Table 1:** Calculated Barriers for Intramolecular Hydrogen-Atom Transfer in the Ground Electronic State

compound	conversion	barrier height^a^ (kJ mol^–1^)
3-thiopyridazine	thione → thiol^b^	128
2-thioquinoline	thione → thiol^b^	127
2-thioimidazole	thione → thiol^c^	161
3-thio-1,2,4-triazole	thione → thiol^c^	161
2-thiobenzothiazole	thione → thiol^d^	152
thioacetamide	thione → thiol^e^	161
thiourea	thione → thiol^e^	167^f^
4-oxopyrimidine	oxo → hydroxy^g^	156
7-azaindole	N(1)H → N(7)H^g^	262

a Calculated at the MP2/6-311++G(2d,p) level.

b See Scheme 2 and refs (14) and (19).

c See Scheme 3 and ref (32).

d See Scheme 4.

e See Scheme 5 and refs (41), (42), and (44)

f See ref (44).

g See Scheme 1 and refs (7) and (28).

### 4.2 UV-Induced Hydrogen-Atom Transfer in 3-Thio-1,2,4-triazole and 2-Thioimidazole: Thione Compounds with a Five-Membered Heterocyclic Ring

**Figure 3:**
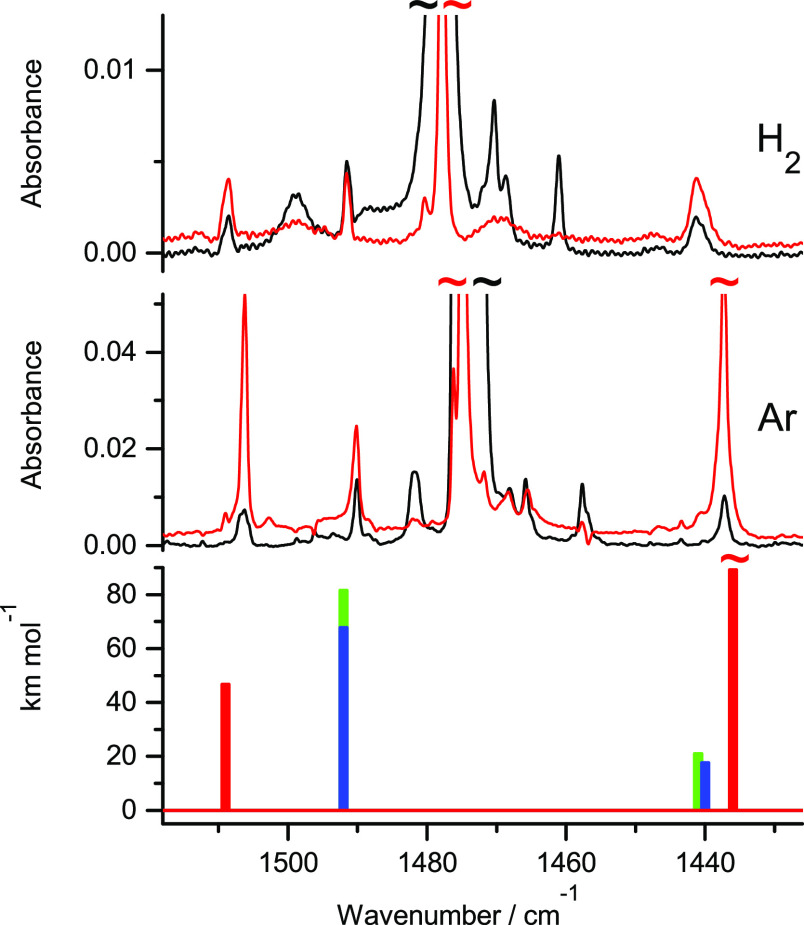
Fragment of the infrared spectrum of 3-thio-1,2,4-triazole monomers: (middle panel) isolated in an Ar matrix; (upper panel) isolated in an n-H_2_ matrix. Black traces represent the spectra recorded directly after deposition of the matrices at 3.5 K; red traces represent the spectra recorded after irradiation of the matrices with UV (λ > 275 nm) light. The experimental spectra are compared with the theoretical spectrum (bottom panel) calculated at the DFT(B3LYP)/6-311++G(2d,p) level for three different thiol isomers of the compound. Red sticks represent the theoretical bands calculated for the thiol form presented in Scheme 3, whereas blue and green sticks represent the theoretical bands computed for the thiol forms presented in Figure S3 of the Supporting Information. The theoretical wavenumbers, calculated within the harmonic approximation, were scaled by 0.98.

Monomers of 3-thio-1,2,4-triazole trapped in low-temperature matrices adopt predominantly the thione tautomeric form. The UV-induced thione → thiol phototautomeric conversion (Scheme 3) of 3-thio-1,2,4-triazole molecules isolated in an Ar matrix was reported in the previous work.^32^ The thiol form of the compound was found to be the main product generated upon UV (λ > 275 nm) excitation of the thione substrate. In the present study, the molecules of 3-thio-1,2,4-triazole isolated in solid n-H_2_ were exposed to UV (λ > 275 nm) light. Detailed analysis of the IR spectra recorded before and after irradiation of 3-thio-1,2,4-triazole isolated in an n-H_2_ matrix revealed that the bands due to the thiol tautomer increased only very slightly upon UV excitation of the compound (Figure 3). This indicates that UV irradiation of 3-thio-1,2,4-triazole isolated in solid n-H_2_ induces only a very small increase of the population of the thiol form. It should be noted that some population of the thiol tautomer (Figure 3) was already present in the matrix before any irradiation.^32^

**Scheme 3:**
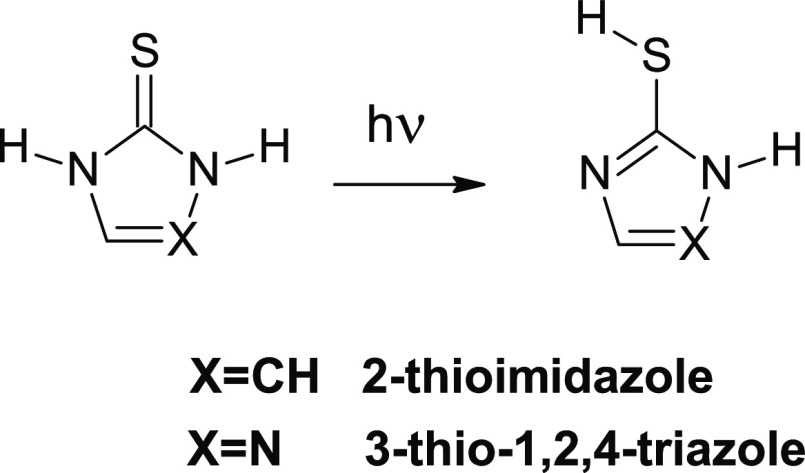
Thione → Thiol Phototautomeric Conversions Observed for Monomers of 2-Thioimidazole and 3-Thio-1,2,4-triazole

2-Thioimidazole is an archetype compound with a five-membered heterocyclic ring and the exocyclic C=S group placed in the direct vicinity of the N–H moiety (Scheme 3). So far, UV-induced thione → thiol phototautomeric reaction was observed only for a methylated derivative of 2-thioimidazole (1-methyl-2-thioimidazole) isolated in Ar matrices.^18^

**Figure 4:**
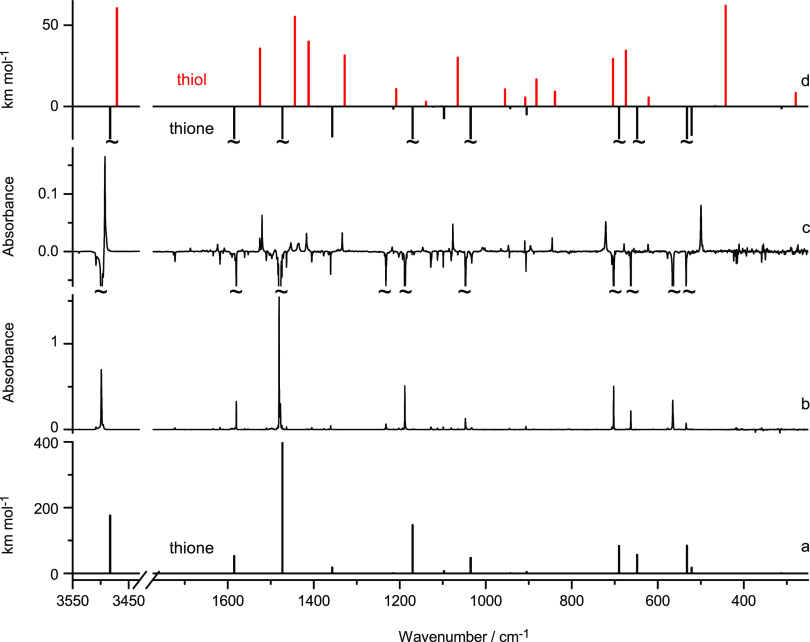
Fragments of the IR spectra of 2-thioimidazole monomers isolated in an Ar matrix: (b) recorded after deposition of the matrix; (c) difference spectrum, the spectrum recorded after UV (λ = 278 nm) irradiation *minus* the spectrum recorded before UV irradiation. Theoretically calculated spectrum of the thione tautomer of the compound is presented in trace (a) as a set of black sticks, and in trace (d) as a set of black truncated sticks pointing down. Theoretically calculated spectrum of the thiol tautomer of 2-thioimidazole is presented in trace (d) as a set of red sticks pointing up. The theoretical wavenumbers, calculated within the harmonic approximation at the DFT(B3LYP)/6-311++G(2d,p) level, were multiplied by 0.95 for wavenumbers higher than 3000 cm^–1^ and by 0.98 for wavenumbers lower than 3000 cm^–1^.

Current investigation shows that 2-thioimidazole monomers deposited from the gas phase into Ar or n-H_2_ matrix exist exclusively in the thione tautomeric form. It was confirmed by a very good match of the IR spectra and the spectrum predicted theoretically (at the DFT(B3LYP)/6-31++G(2d,p) level) for the thione tautomer of the compound (see Figure 4, Figure S4, and Table S1 in the Supporting Information).

**Figure 5:**
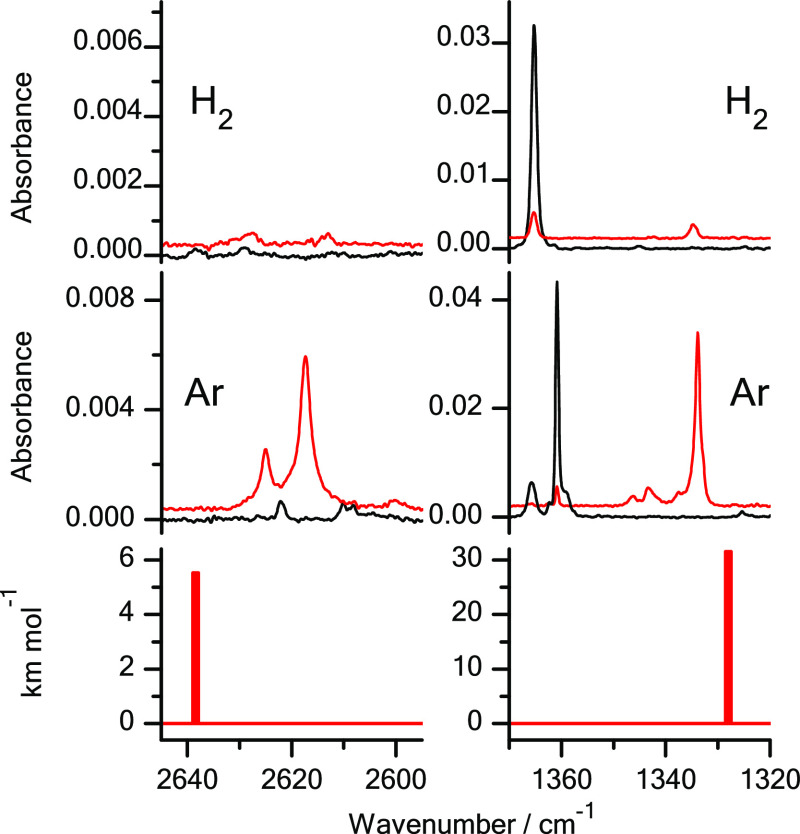
Fragments of the infrared spectra of 2-thioimidazole monomers: (middle panel) isolated in an Ar matrix; (upper panel) isolated in an n-H_2_ matrix. Black traces represent the spectra recorded directly after deposition of the matrices at 3.5 K; red traces represent the spectra recorded after irradiation of the matrices with UV (λ = 278 nm) light. The experimental spectra are compared with the theoretical spectrum (bottom panel) calculated at the DFT(B3LYP)/6-311++G(2d,p) level for the thiol tautomer of the compound. The theoretical wavenumbers, calculated within the harmonic approximation, were multiplied by 0.98.

When the compound isolated in solid Ar was irradiated with UV (λ = 278 nm) light, the photoinduced thione → thiol conversion was clearly observed (see Figures 4 and 5). Upon such irradiation, the IR bands belonging to the spectrum of the thione tautomer significantly decreased, whereas a new spectrum consisting of low-intensity bands emerged. In this new spectrum, the expected bands due to νNH and νSH vibrations were found at 3493 and 2617 cm^–1^, respectively. Also, the general pattern of the IR bands due to the photoproduced species is well reproduced by the pattern of bands theoretically predicted for the thiol form of 2-thioimidazole (Figure 4). Hence, for 2-thioimidazole isolated in an Ar matrix, photogeneration of the thiol form as the dominating product was positively confirmed. Note that, in general, the IR absorption bands due to thiol forms are weaker than those of thione forms;^14,15,18−20^ the sum of calculated absolute intensities of all IR bands of the thiol form is several times lower than the respective sum calculated for the thione form.

The photochemical behavior of 2-thioimidazole isolated in solid n-H_2_ was different. UV (λ = 278 nm) irradiation of an n-H_2_ matrix containing monomers of 2-thioimidazole did not lead to generation of the thiol form as the main photoproduced species. After UV irradiation of the n-H_2_ matrix, only a few very low-intensity IR bands appeared close to the spectral positions of IR bands of the thiol photoproduct generated from 2-thioimidazole isolated in solid Ar (Figure 5). This indicates that upon UV (λ = 278 nm) excitation only a very small fraction of the initial thione tautomer of 2-thioimidazole was converted to the thiol product. The majority of the reactant (the thione form of 2-thioimidazole) was photochemically converted to unidentified species.

As far as the UV-induced thione → thiol hydrogen-atom transfer is concerned, the patterns of the photochemical behavior of matrix-isolated 3-thio-1,2,4-triazole and 2-thioimidazole are quite similar to each other. For both thione compounds with a five-membered heterocyclic ring consisting only of nitrogen and carbon atoms, the thione → thiol phototautomeric reaction occurs in Ar matrices, leading to the generation of the thiol tautomer as the main photoproduct. However, in n-H_2_ matrices, only trace amounts of thiol tautomers were produced upon UV irradiation. This behavior is quite different from that observed for the compounds with a six-membered heterocyclic ring, such as 3-thiopyridazine and 2-thioquinoline.

The calculated barriers for the thione → thiol tautomeric conversion in the ground electronic state of 2-thioimidazole and 3-thio-1,2,4-triazole (Table 1) are significantly higher (161 kJ mol^–1^ for each of compounds) than those computed for thione heterocycles with a six-membered ring (127–128 kJ mol^–1^, see Section 4.1). Different heights of the barriers are related to different geometries of the molecules in question. The reason for the barriers as high as 161 kJ mol^–1^ is the rigid five-membered ring that makes the angles in the thiolactam H–N–C=S fragment of the thione forms of 2-thioimidazole (and 3-thio-1,2,4-triazole) as large as ∠SCN = 128° (129°) and ∠CNH = 121° (122°). Consequently, the (N)H···S hydrogen–sulfur distance in the thiolactam fragment of the thione tautomer gets as large as 2.96 Å (2-thioimidazole) and 3.00 Å (3-thio-1,2,4-triazole).On the other hand, for 3-thiopyridazine (sixmembered ring) the respective geometrical parameters are as follows: ∠SCN = 121°, ∠CNH = 116°, and (N)H···S distance is equal to 2.71 Å.

### 4.3 UV-Induced Hydrogen-Atom Transfer in 2-Thiobenzothiazole: The Thione Compound with a Five-Membered Thiazole Ring that Contains an Endocyclic Sulfur Atom

UV-induced phototautomerism of 2-thiobenzothiazole has not been investigated so far. In the current work, the photochemical behavior of monomers of this compound isolated in Ar and n-H_2_ matrices was studied for the first time. This study demonstrated that, after deposition of low-temperature matrices, the trapped monomers of the compound adopt exclusively the thione tautomeric form (Figure S5 and Table S2 in the Supporting Information).

**Figure 6:**
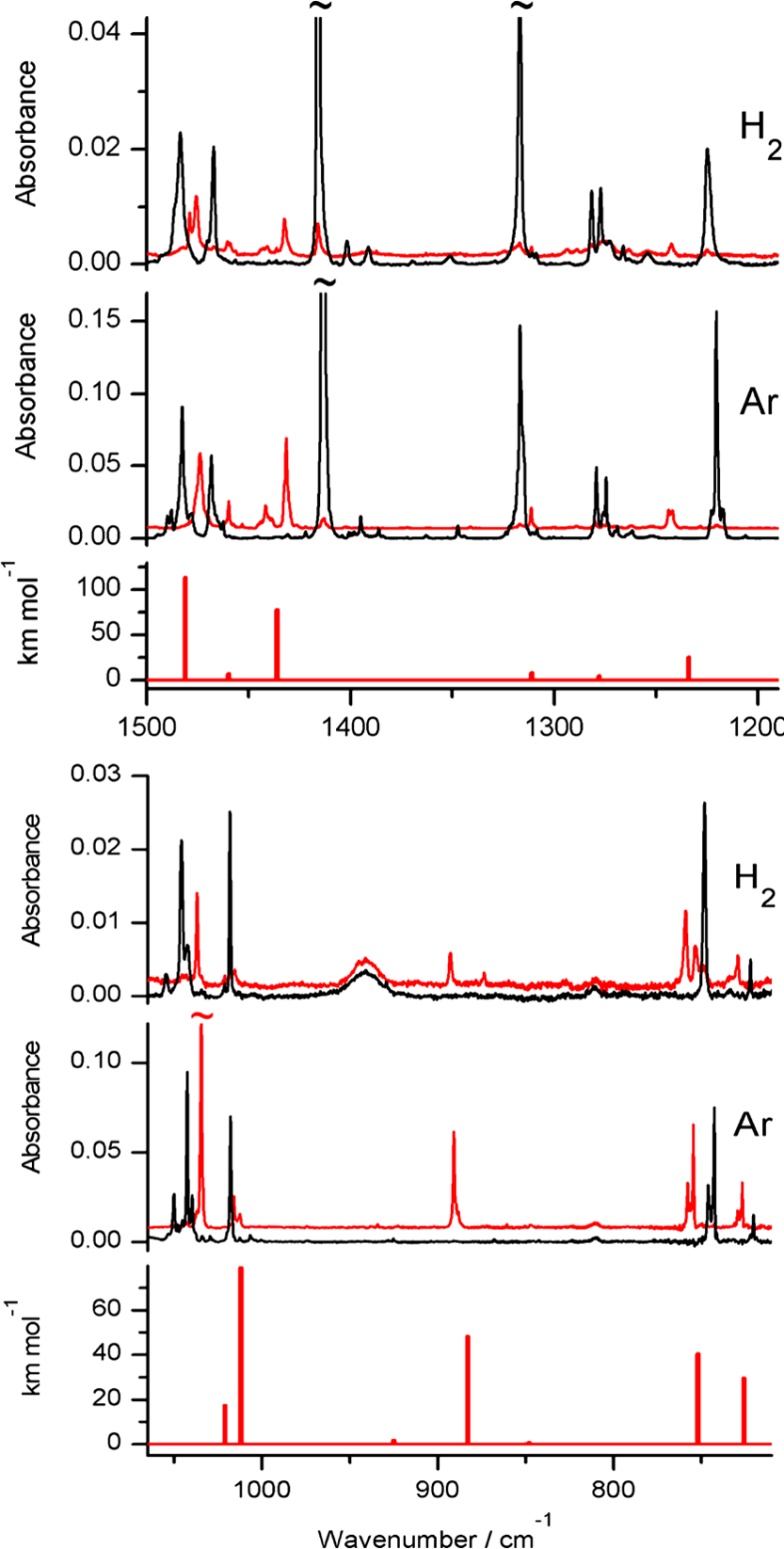
Fragments of the infrared spectra of 2-thiobenzothiazole monomers: (middle panels) isolated in an Ar matrix; (top panels) isolated in an n-H_2_ matrix. Black traces represent the spectra recorded directly after deposition of the matrices at 3.5 K; red traces represent the spectra recorded after irradiation of the matrices with UV (λ = 305 nm) light. The experimental spectra are compared with the theoretical spectrum (bottom panel) calculated at the DFT(B3LYP)/6-311++G(2d,p) level for the thiol tautomer of the compound. The theoretical wavenumbers, calculated within the harmonic approximation, were scaled by 0.98.

**Scheme 4:**
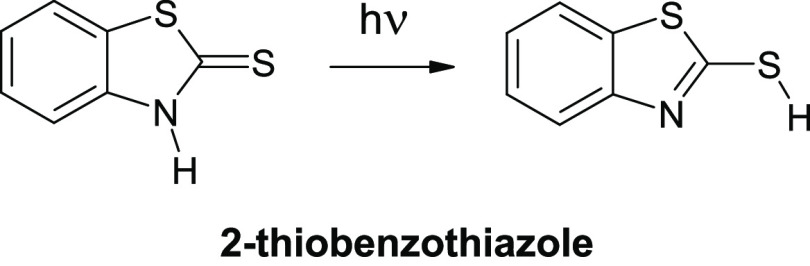
Thione → Thiol Phototautomeric Conversion Observed for Monomers of 2-Thiobenzothiazole

Upon irradiation of an Ar matrix with UV (λ = 305 nm) light, the thione monomers of 2-thiobenzothiazole efficiently transform into the thiol tautomeric form of the compound (Scheme 4 and Figure 6). The thiol tautomer is also photoproduced upon UV (λ = 305 nm) irradiation of the compound isolated in an n-H_2_ matrix (Figure 6), but the yield of this photoprocess is ca. 3 times lower than the yield of analogous phototautomerism occurring for the compound isolated in solid Ar (Figure S6 in the Supporting Information). Nevertheless, the amount of the thiol form of 2-thiobenzothiazole photogenerated from the thione precursor trapped in an n-H_2_ matrix is clearly higher than the tiny quantities of the thiol products photogenerated from the thione tautomers of 2-thioimidazole or 3-thio-1,2,4-triazole isolated in n-H_2_ matrices. Although the transforming H–N–C=S fragment of the 2-thiobenzothiazole molecule is a part of the five-membered thiazole ring, the photochemical behavior of the compound isolated in solid n-H_2_ is more similar to that observed for compounds with a six-membered heterocyclic ring than for compounds with a five-membered heterocyclic ring. This may be related to the lower barrier for the ground-state thione → thiol tautomerism in 2-thiobenzothiazole (152 kJ mol^–1^, Table 1) in comparison to the analogous barriers in 2-thioimidazole or 3-thio-1,2,4-triazole (161 kJ mol^–1^ for each of these compounds). This, in turn, is the consequence of the presence of two C–S single bonds in the thiazole ring. Such C–S bonds are significantly longer (1.75–1.77 Å) than the C–N, N–N, or C–C single bonds (1.36–1.38 Å) present in the structures of imidazole or triazole rings. Two long C–S bonds make the geometry of the thiazole ring somewhat similar to the geometry of a six-membered ring. As the result, the (N)H···S hydrogen–sulfur distance in the H–N–C=S fragment of 2-thiobenzothiazole molecule is shorter (2.86 Å) than the analogous distance for 2-thioimidazole (2.96 Å) or for 3-thio-1,2,4-triazole (3.00 Å).

### 4.4 UV-Induced Thione → Thiol Hydrogen-Atom Transfer in Thiourea and Thioacetamide

**Scheme 5:**
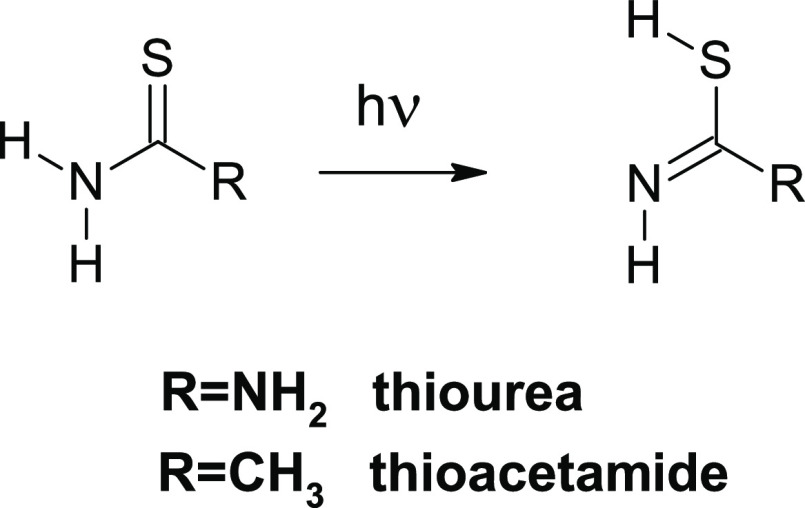
Thione → Thiol Phototautomeric Conversions Observed for Monomers of Thiourea and Thioacetamide

Previous studies on small thioamide molecules, thiourea and thioacetamide, showed that monomers of these species isolated in Ar matrices adopt solely thione tautomeric forms.^40,41^ For monomers of both compounds isolated in Ar matrices and irradiated with UV light, the phototautomeric thione → thiol conversion (Scheme 5) was previously observed.^41−43^ More recently, we studied thiourea isolated in Ne, H_2_, and D_2_ low-temperature matrices.^44^ In that work, we demonstrated that also in these matrices the thione tautomer of thiourea is exclusively populated prior to any irradiation. We have also shown that, upon excitation with UV (λ > 270 nm) light, this thione tautomer (isolated in Ne, H_2_, or D_2_ matrices) undergoes phototautomeric reaction and converts into the thiol form.^44^ For thiourea isolated in solid D_2_, no spectral indications of a spontaneous or UV-induced H ↔ D isotopic exchange between the matrix material and the isolated compound were detected at any stage of the observed phototautomeric process. These experimental observations are similar to those concerning 4-pyrimidinone isolated in solid D_2_ and excited with UV light.^31^ This demonstrates that the D_2_ matrix environment does not act as a reagent directly involved in the phototautomeric process.

**Figure 7:**
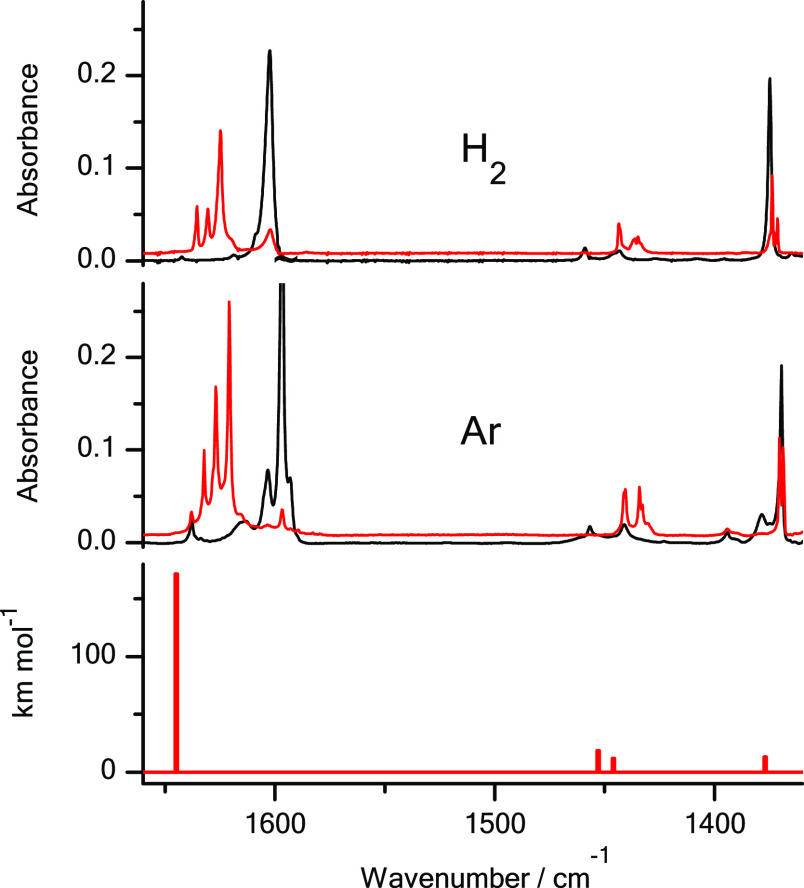
Fragments of the infrared spectra of 2-thioacetamide monomers: (middle panel) isolated in an Ar matrix; (top panel) isolated in an n-H_2_ matrix. Black traces represent the spectra recorded directly after deposition of the matrices at 3.5 K; red traces represent the spectra recorded after irradiation of the matrices with UV (λ = 278 nm) light. The experimental spectra are compared with the theoretical spectrum (bottom panel) calculated at the DFT(B3LYP)/6-311++G(2d,p) level for the thiol tautomer of the compound. The theoretical wavenumbers, calculated within the harmonic approximation, were scaled by 0.98.

**Figure 8:**
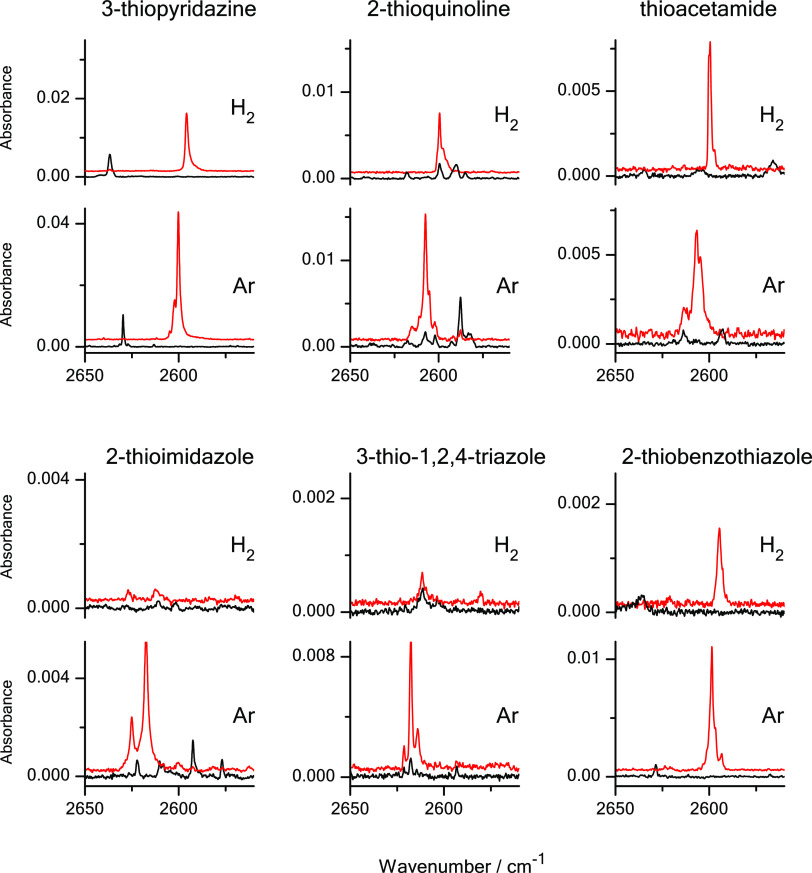
Spectral changes in the region where the bands due to the stretching vibrations of SH group (νSH) are found in the infrared spectra of 3-thiopyridazine, 2-thioquinoline, thioacetamide, 2-thioimidazole, 3-thio-1,2,4-triazole, and 2-thiobenzothiazole isolated Ar and n-H_2_ matrices. Black traces represent the spectra recorded directly after deposition of the matrices at 3.5 K; red traces represent the spectra recorded after irradiation of the matrices with UV (λ = 305 or 278 nm) light.

In the current work, we investigated the photochemical behavior of thioacetamide monomers isolated in solid n-H_2_. In this study, we observed that also in n-H_2_ matrices monomers of thioacetamide adopt exclusively the thione tautomeric form. Upon UV (λ = 278 nm) irradiation, this thione form converts to thiol tautomer (Figure 7). The thiol tautomer of thioacetamide was efficiently photoproduced in both Ar and n-H_2_ matrices (see the effects presented in Figures 7 and 8). The yield of the thiol photoproduct generated in n-H_2_ matrices can be assessed as 45% of the yield of the thiol form photoproduced in Ar matrices.

The barriers for the ground-electronic-state thione → thiol tautomeric conversion theoretically assessed for thiourea and thioacetamide are similar (or slightly higher) to the energy barriers computed for 2-thioimidazole or 3-thio-1,2,4-triazole (Table 1). However, the efficiency of photogeneration of the thiol forms of molecules trapped in n-H_2_ matrices was clearly much higher for thioacetamide than it was for 2-thioimidazole and 3-thio-1,2,4-triazole. For thioacetamide, the amount of the thiol form photoproduced in solid n-H_2_ was comparable to the amount of the thiol form photogenerated in a solid Ar environment (Figures 7 and 8), whereas for 2-thioimidazole and 3-thio-1,2,4-triazole isolated in solid n-H_2_, only some barely detectable traces of the thiol forms were photoproduced. The obvious difference between heterocyclic thione compounds and simple thioamides is the rigidity of the molecular frame (heterocyclic ring) present in the structure of 2-thioimidazole or 3-thio-1,2,4-triazole and flexibility of the molecular frame of thiourea and thioacetamide. It seems that rigidity or flexibility of the molecule may be one of the factors that determine different photochemical behavior of simple thioamides (flexible) and heterocyclic compounds (rigid) isolated in solid n-H_2_ and excited with UV light.

## 5 Discussion

Phototautomeric conversions, examples of which were presented in the previous sections, are typical phototransformations commonly occurring for monomeric heterocyclic compounds containing lactam or thiolactam group and isolated in Ar, Xe, Ne, or N_2_ low-temperature matrices. The mechanism of these photoprocesses is still not completely clear.

Similarly typical is the UV-induced hydrogen-atom detachment observed for many heterocyclic molecules in the gas phase (seeded in supersonic jets).^45^ It was experimentally demonstrated that upon UV excitation of gaseous pyrrole,^46,47^ imidazole,^48^ phenol,^49^ or indole^50^ seeded in the supersonic expansions, two types of hydrogen atoms detach from the N–H or O–H moieties. Hydrogen atoms of one type are slow, and they detach from high vibrational levels of the ground electronic state. Such high vibrational levels are populated during energy dissipation, which follows UV excitation of the molecule. Another type concerns fast hydrogen atoms, which gain momentum when they move away from the molecule on the surface of the repulsive ^1^πσ* excited electronic state. In the system evolving on the surface of the ^1^πσ* excited state, the potential electronic energy is converted into kinetic energy of the dissociating hydrogen atom.^50,51^

Detachment of fast hydrogen atoms from UV-excited heterocyclic molecules was theoretically predicted by Sobolewski et al.^51^ Hydrogen-atom detachment on the potential-energy surfaces of the repulsive ^1^πσ states was postulated as a new paradigm in the photochemistry of heterocyclic compounds. Based on these ideas, a theoretical model (photoinduced dissociation association, PIDA) was formulated to describe the mechanism of phototautomeric reaction in 4-oxopyrimidine.^35^ According to this model, the first stage of the phototautomeric transformation should concern a detachment of the hydrogen atom on the potential energy surface of the repulsive ^1^πσ* excited state. This movement continues toward the intersection seam of the ^1^πσ* state with the ground electronic state. The second stage of the process should concern a movement of the hydrogen atom on the surface of the ground electronic state toward one of the minima corresponding to the oxo or hydroxy tautomeric form. Since the intersection seam of the ^1^πσ* and the ground states is located at some distance from the equilibrium position of the hydrogen atom attached to the molecule, the environment may affect the progress of phototransformation involving detachment of the hydrogen atom followed by its reassociation to the matrix-isolated molecule.

In the PIDA model, the fast dissociating hydrogen atom must change the direction of its movement back toward association with the molecule. The only turning point, we may imagine, is at the walls of the matrix cage. After a collision with a heavy Ar (Xe, Ne) atom in the rigid crystalline lattice of the matrix, the direction of hydrogen-atom momentum should reverse, allowing association of the hydrogen atom with the remaining main body of the molecule. For the molecules isolated in a solid H_2_ matrix, the case may be different. It is known^33,34^ that solid hydrogen, which is composed of H_2_ molecules, is much “softer” than Ar crystal. The collision of the fast dissociating hydrogen atom with the light H_2_ molecule in the soft matrix environment may lead to considerable loss of the hydrogen-atom velocity and, hence, significantly limit the possibility of hydrogen-atom reassociation with the remaining part of the molecule.

The PIDA mechanism seems to be the best currently available model of the experimentally observed phototautomeric processes, but having in mind the observation of slow hydrogen atoms dissociating from the ground electronic state of molecules in the gas phase, we postulate that the change of the tautomeric form (by hydrogen-atom transfer) can also take place during dissipation of excitation energy when molecules are in very high vibrational states of the ground electronic state. In the high vibrational states of the molecule, labile hydrogen atoms (such as those from the N–H groups) may achieve sufficient energy to cross the barrier dividing the minima of different tautomers. Such hydrogen atoms can crawl from one heteroatom to another, without any detachment from the molecule. The possibility of intramolecular hydrogen-atom transfer induced by excitation of higher vibrational states of the ground electronic state has recently been experimentally proven.^52^ It was demonstrated that near-IR excitation of thiotropolone leads to the transformation of the thio-hydroxy tautomer into the oxo-thiol tautomeric form. This transformation occurs by a hydrogen-atom shift from the oxygen atom to the vicinal sulfur atom of the thiotropolone molecule.^52^ This mechanism may be operative especially for cases where the energy barriers for tautomerization in the ground electronic state are relatively low. For molecules, where the tautomers are separated by very high barriers, the PIDA mechanism involving dissociation of a hydrogen atom, followed by its association with another heteroatom, seems to be more adequate.

For the photoinduced tautomeric transformation to occur by crossing the barrier in the vibrational excited levels of the ground electronic state, the height of the barrier is of crucial importance. The heights of the barriers for the ground-electronic-state tautomerization were theoretically assessed [at the MP2/6-311++G(2d,p) and DFT(B3LYP)/6-311++G(2d,p) levels] not only for the thione compounds experimentally investigated within the current work, but also for such compounds as 7-azaindole, 4-oxopyrimidine, and 6-hydroxy-4-oxopyrimidine (see Tables 1 and S3 in the Supporting Information).

The highest barrier (262 kJ mol^–1^) was computed for 7-azaindole, where the hydrogen atom is transferred from N(1) to quite remote N(7) atom (Scheme 1). Such a very high barrier should preclude any change of tautomeric form in the ground electronic state. For 7-azaindole, the UV-induced N(1) → N(7) phototautomeric reaction which occurred in a solid Ar environment did not occur at all in solid n-H_2_.^28^

The lowest barriers (127–128 kJ mol^–1^) were predicted for 3-thiopyridazine and 2-thioquinoline, the compounds with the thiolactam group directly attached to a six-membered heterocyclic ring. For these species isolated in n-H_2_ matrices, the yield of the thione → thiol conversion was 25–50% of the yield of the analogous phototautomeric transformation observed for the same compounds isolated in solid Ar (Figures 1, 2, 8 and Figures S1, S2 in the Supporting Information). It seems possible that for such species, in their high vibrational levels of the ground electronic state, the energy of the hydrogen atom may be sufficient for crossing the barrier for tautomerization. Such processes, not involving dissociation of hydrogen atoms, should occur in a similar way in both rigid Ar matrices and in soft H_2_ matrices. According to the proposed model, the hydrogen-atom transfer in the excited vibrational states of the ground electronic state should be responsible for phototautomerism observed for molecules isolated in H_2_ matrices. In rigid matrices (e.g., in solid Ar), this mechanism of phototautomerization should also be operative, but usually accompanied by the dominating phototautomerism following the PIDA scheme.

For the species described above, where the barriers are either very high or relatively low, the height of the barrier correlates well with the ability of molecules isolated in n-H_2_ matrices to undergo UV-induced hydrogen-atom transfer. For the remaining compounds, with the barriers for tautomerization of intermediate heights (151–167 kJ mol^–1^, Table 1), the correlation is not so straightforward. Various patterns of dependence of the UV-induced thione → thiol or oxo → hydroxy conversions on the low-temperature environment (Ar or H_2_) were observed for these molecules.

2-Thioimidazole and 3-thio-1,2,4-triazole, the thione compounds with a five-membered heterocyclic ring, do efficiently phototautomerize in Ar matrices. However, in a solid n-H_2_ environment, the UV-induced thione → thiol conversion generates only trace amounts of the thiol products (Figures 3, 4, and 8). Probably, the less favorable geometry of the H–N–C=S fragment (the H to S distance as large as 2.96–3.00 Å), as well as the relatively high barrier for the thione → thiol tautomerization in the ground electronic state, is responsible for the low efficiency of the phototautomeric transformation observed for the species isolated in solid n-H_2_.

The compounds with a six-membered heterocyclic ring and the C=O group directly attached to it (4-oxopyrimidine and 6-hydroxy-4-oxopyrimidine) do readily phototransform from the oxo to the hydroxy form. However, this photoinduced oxo → hydroxy conversion occurred only for the species isolated in Ar matrices; in a solid n-H_2_ environment, the oxo → hydroxy phototransformation did not occur at all.^30,31^ This type of photochemical behavior must be related to the presence of oxygen atom in the C=O group (which is the target for the transferring hydrogen atom) because in thione compounds (3-thiopyridazine and 2-thioquinoline), having an analogous six-membered heterocyclic ring but the C=S group as a target of hydrogen shift, the thione → thiol phototransformation occurred in both Ar and n-H_2_ low-temperature environments.

Although for thioacetamide and thiourea, the calculated barriers for the ground-electronic-state thione → thiol tautomerizations are quite high (161 and 167 kJ mol^–1^, respectively, Table 1), these compounds readily phototautomerize not only in a solid Ar environment, but also in solid n-H_2_ matrices (Figures 7, 8 and ref (44)). Evidently, the flexible frame of thioacetamide or thiourea molecules facilitates the thione → thiol hydrogen-atom transfer better than the rigid molecular frame of such compounds as 2-thioimidazole or 3-thio-1,2,4-triazole.

## 6 Conclusions

The investigations on UV-induced hydrogen-atom-transfer processes, carried out so far for such compounds as 7-azaindole, 4-oxopyrimidine, and 6-hydroxy-4-oxopyrimidine^28,30,31^ isolated in Ar and n-H_2_ low-temperature matrices, might have led to the conclusion that the replacement of solid argon by a solid-hydrogen environment inevitably precludes phototautomeric transformations. The current study, performed for a number of thione compounds with the H–N–C=S moiety in the structure, demonstrated that this is not always the case. We have shown that phototautomeric thione → thiol transformations depend on the matrix material (Ar or n-H_2_) in a variety of manners. For compounds with relatively high barriers separating (on the potential-energy surface) the minima of different tautomers, the phototautomeric reaction occurred only for the molecules isolated in solid Ar. Nearly no phototautomeric conversion was observed for molecules of these compounds isolated in solid n-H_2_ matrices. In such species, high barriers may preclude any change of the tautomeric form in the ground electronic state and only phototautomerism by the dissociation–association (PIDA) mechanism may be possible. The latter mechanism seems to be operative only for molecules trapped in rigid cages of Ar (Xe or N_2_) matrices.

For compounds (such as 3-thiopyridazine and 2-thioquinoline), where the barriers separating the potential-energy minima of different tautomers are lower, UV-induced change of the tautomeric form was observed not only for the molecules isolated in solid Ar, but also (though with lower 25–50% efficiency) for molecules isolated in solid hydrogen. We suppose that the change of the tautomeric form of these molecules trapped in solid n-H_2_ occurs in a strictly intramolecular way, by crossing the relatively low barrier during dissipation of the excitation energy, when the molecules are in high vibrational levels of the ground electronic state. In such highly excited vibrational states, the hydrogen atom may migrate by “crawling” over the low barrier to the other potential-energy minimum. The latter mechanism is more probable for thione compounds, where the distance for the hydrogen shift is smaller than the distance in the analogous oxo molecules.

The current systematic study on the effect of the matrix environment (solid Ar or solid n-H_2_) on the efficiency of the investigated phototautomerizations resulted in several unexpected findings and provided a new information about the mechanism(s) of the investigated photoprocesses.

## Supporting Information Available

Analysis of the progress of the thione → thiol phototautomeric reaction observed for monomers of 3-thiopyridazine, 2-thioquinoline, and 2-thiobenzothiazole isolated in Ar and n-H_2_ matrices; the infrared spectra of 2-thioimidazole and 2-thiobenzothiazole isolated in Ar and n-H_2_ matrices and the assignment of these spectra; and structures of three thiol isomers of 3-thio-1,2,4-triazole (PDF)
