# Supplementary material for: Effect of a Solid-Hydrogen Environment on UV-Induced Hydrogen-Atom Transfer in Matrix-Isolated Heterocyclic Thione Compounds
Source: J Phys Chem A. 2021 Aug 18;125(34):7437–48. doi: 10.1021/acs.jpca.1c05538 (PMC8419844; doi:10.1021/acs.jpca.1c05538)
Supplement: Supplementary file 1 — jp1c05538_si_001.pdf [file jp1c05538_si_001.pdf]

Effect of Solid-Hydrogen Environment on UV-induced Hydrogen-Atom Transfer in Matrix-Isolated Heterocyclic Thione Compounds

Hanna Rostkowska, Anna Luchowska, Leszek Lapinski, and Maciej J. Nowak

Institute of Physics, Polish Academy of Sciences, Al. Lotnikow 32/46, 02-668 Warsaw, Poland

Supporting Information

Contents:

Figure S1	The comparison of the progress of the thione → thiol phototautomeric reaction in monomers of 3-thiopyridazine isolated in Ar and n-H_2 matrices.	p. S3
Figure S2	The comparison of the progress of the thione → thiol phototautomeric reaction in monomers of 2-thioquinoline isolated in Ar and n-H_2 matrices.	p. S4
Note S1	Remarks concerning the feasibility of reliable estimation of quantum yield of phototransformations occurring in matrix-isolated molecules.	p. S4
Figure S3	Thiol isomers of 3-thio-1,2,4-triazole generated upon UV(λ > 275 nm) irradiation of the monomers of the compound, isolated in Ar or n-H_2 matrices.	p. S5
Figure S4	Fragments of the IR spectra of 2-thioimidazole monomers isolated in: n-H_2 and Ar matrices. The spectra are juxtaposed with the theoretical spectrum calculated, at the DFT(B3LYP)/6-311++G(2d,p) level, for the thione tautomer of the compound.	p. S5
Table S1	Approximate assignment of the absorption bands, observed in the IR spectrum of monomers of 2-thioimidazole isolated in Ar and n-H_2 matrices, to the normal modes calculated, at the DFT(B3LYP)/6-311++G(2d,p) level of theory, for the thione tautomer of the compound.	p. S6
Figure S5	Fragments of the IR spectra of 2-thiobenzothiazole monomers isolated in: n-H_2 and Ar matrices; juxtaposed with the theoretical spectrum calculated, at the DFT(B3LYP)/6-311++G(2d,p) level, for the thione tautomer of the compound.	p. S7
Figure S6	The comparison of the progress of the thione → thiol phototautomeric reaction in monomers of 2-thiobenzothiazole isolated in Ar and n-H_2 matrices.	p. S7
Table S2	Approximate assignment of the absorption bands, observed in the IR spectrum of monomers of 2-thiobenzothiazole isolated in Ar and n-H_2 matrices, to the normal modes calculated, at the DFT(B3LYP)/6-311++G(2d,p) level of theory, for the thione tautomer of the compound.	p. S8-S9
Table S3	Structures of the tautomeric forms of the studied compounds and the height of the barrier for the thione→thiol, oxo→hydroxy or N(1)H→N(7)H transformation, calculated at the DFT(B3LYP)/6-311++G(2d,p) level and at the MP2/6-311++G(2d,p) level.	p. S10-S11

The estimation of relative amounts of photoproduced thiol tautomers of the studied compounds isolated in an Ar and H_2 matrices is not a straightforward task because the relative intensities of the corresponding absorption bands in the spectra of the monomers isolated in Ar and H_2 matrices are usually different. Therefore we assumed that the amount of absorbing species in Ar and H_2 matrices is proportional to the average intensity of several, most prominent absorption bands present in the spectrum. The average intensities were scaled in such a way that the average intensities of the bands due to molecules in thione form in the spectrum obtained before UV irradiation in Ar and H_2 matrices are equal to 1. Such procedure was applied for Figures S1, S2 and S6.

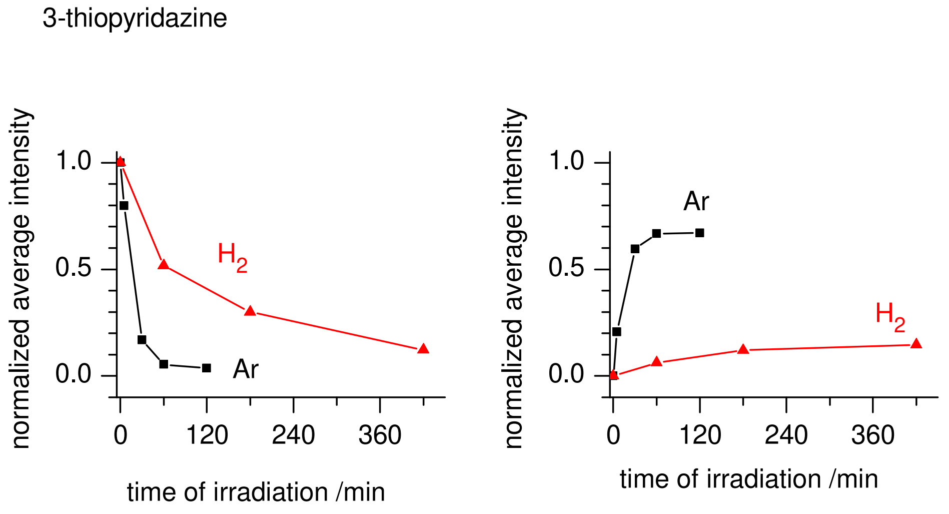

Decay of the thione tautomer during UV (305 nm) irradiation

Formation of the thiol tautomer during UV (305 nm) irradiation

Figure S1. The comparison of the progress of the thione → thiol phototautomeric reaction in monomers of 3-thiopyridazine isolated in Ar (black traces) and n-H_2 (red traces) matrices.

As it is usually the case, the average intensity of the IR bands in the spectrum of the thiol product was much smaller than the average intensity of the bands in the spectrum of the thione reactant. The absolute intensities of IR bands in the spectra of thiol forms are usually lower (as average) than the absolute intensities of the bands in the spectra of thione tautomers.

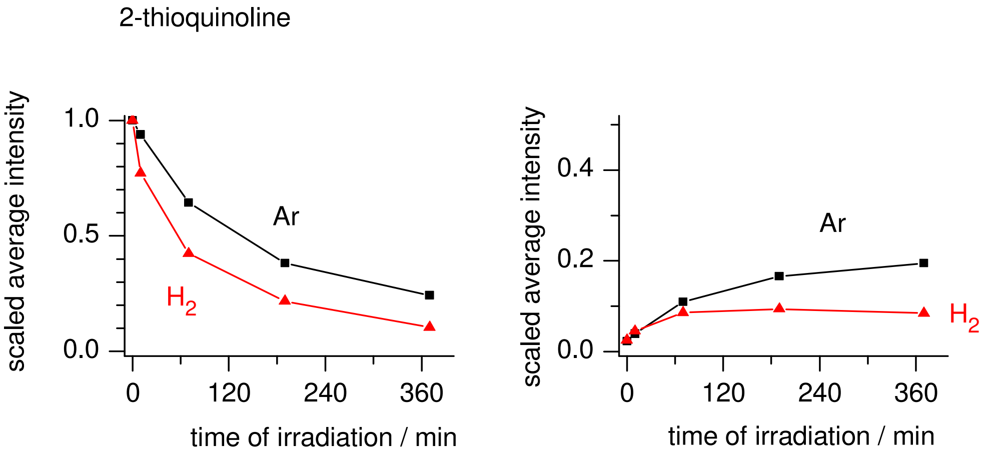

Decay of the thione tautomer during UV (305 nm) irradiation

Formation of the thiol tautomer during UV (305 nm) irradiation

Figure S2. The comparison of the progress of the thione → thiol phototautomeric reaction in monomers of 2-thioquinoline isolated in Ar (black traces) and n-H_2 (red traces) matrices.

Note S1. Reliable measurement of quantum yields of light-induced processes occurring in matrix-isolated compounds is extremely difficult. That is why quantum yields of phototransformations of matrix-isolated species are extremely rarely measured. The reliability of the very few attempts carried out so far can be easily questioned. The reason for this is the fact that a low-temperature matrix is a very dispersive medium, especially for the UV light. Hence, the irradiation conditions of the molecules trapped in the layers closer to the surface are very different from the conditions of irradiation of the molecules trapped in deeper layers of the matrix.

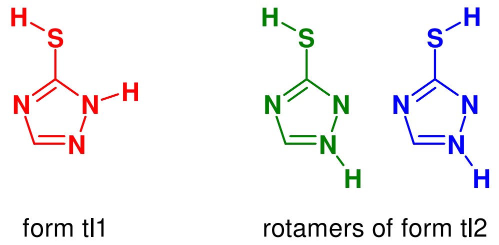

Figure S3. Thiol isomers of 3-thio-1,2,4-triazole generated upon UV (λ > 275 nm) irradiation of the monomers of the compound, isolated in Ar or n-H_2 matrices. Colors of the structures correspond to the colors of the sticks in the bottom panel of Fig. 6, where fragments of the theoretically calculated IR spectra of the three isomers are presented.

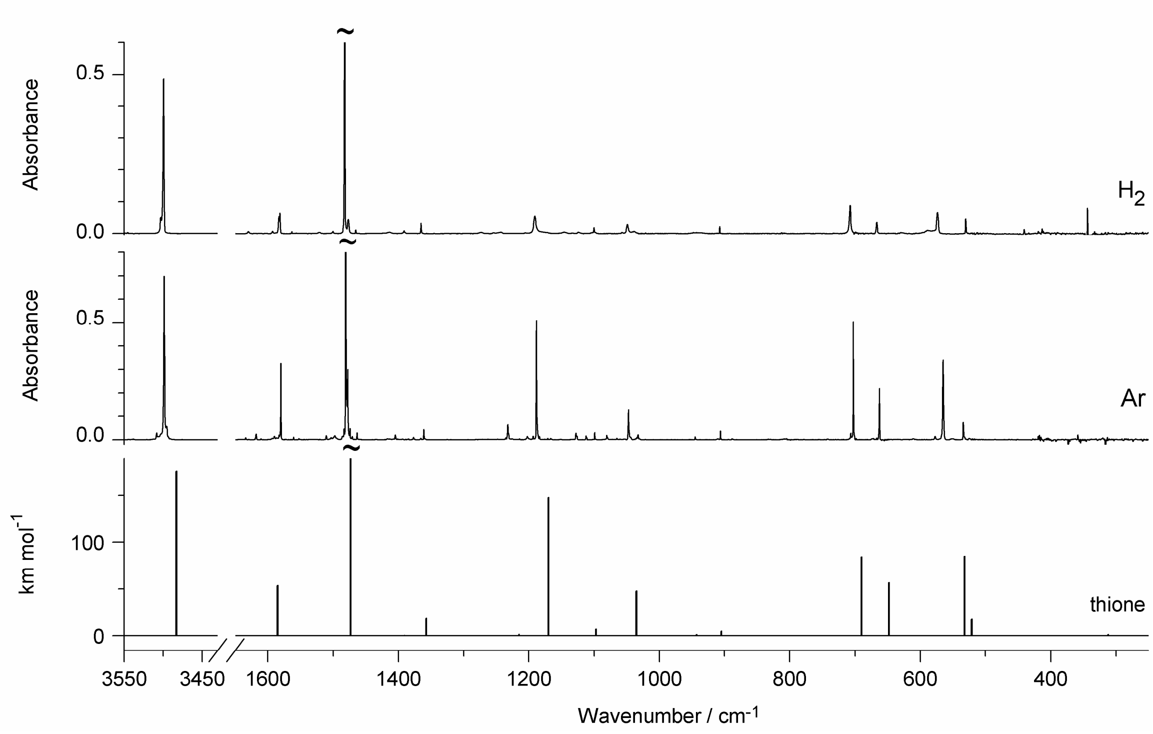

Figure S4. Fragments of the IR spectra of 2-thioimidazole monomers isolated in: (upper panel) n-H_2 and (middle panel) Ar matrices. The spectra are juxtaposed with the theoretical spectrum (bottom panel) calculated at DFT(B3LYP)/6-311++G(2d,p) level for the thione tautomer of the compound. The theoretical wavenumbers, calculated within the harmonic approximation, higher than 3000 cm^{-1} were multiplied by 0.95, whereas the wavenumbers lower than 3000 cm^{-1} were multiplied by 0.98.

Table S1. Approximate assignment of the absorption bands, observed in the IR spectrum of monomers of 2-thioimidazole isolated in Ar and n-H_2 matrices, to the normal modes calculated, at the DFT(B3LYP)/6-311++G(2d,p) level of theory, for the thione tautomer of the compound.

Experiment				Calculation		
Ar matrix		n-H_2 matrix		DFT(B3LYP) /6-311++G(2d,p)		Approximate description
$\tilde{\nu}$ cm^{-1}	I *rel.*	$\tilde{\nu}$ cm^{-1}	I *rel.*	$\tilde{\nu}$ [a] cm^{-1}	A^{th} km mol^{-1}	
3499, 3498sh	187	3501sh, 3499	191	3485	0.001	ν NH
				3483	176	ν NH
3193	1	3186	1	3128	0.3	ν CH
3158	6	3157	5	3110	3	ν CH
1580	49	1583, 1581	57	1585	53	ν C=C
1481, 1477	380	1482, 1477	399	1473	397	β_s NH, ν_s CN
1361	9	1365	15	1357	19	β_a NH, ν_a CN
1189	106	1191	106	1170	148	ν_s CN, ν C=S
1099	6	1100	13	1097	7	ν_s CN, β_s NH
1048	39	1049	55	1035	48	ν_a CN, β_a CH
945	2	947	1	943	1	β R, β_s CH
907	6	907	7	905	5	β R
703	88	708	87	690	84	γ CH
662	36	667	24	648	57	γ CH
565	120	574	79	532	85	γ NH
534	18	530	22	521	18	ν C=S

[a] The theoretical wavenumbers higher than 3000 cm^{-1} were scaled by a factor of 0.95, whereas those lower than 3000 cm^{-1} were scaled by 0.98.
ν stretching, β bending, τ torsion, γ wagging, β R bending of the ring
ν_s symmetric stretching, β_s symmetric bending,
ν_a antisymmetric stretching, β_a antisymmetric bending
The strongest bands are underlined; sh - shoulder

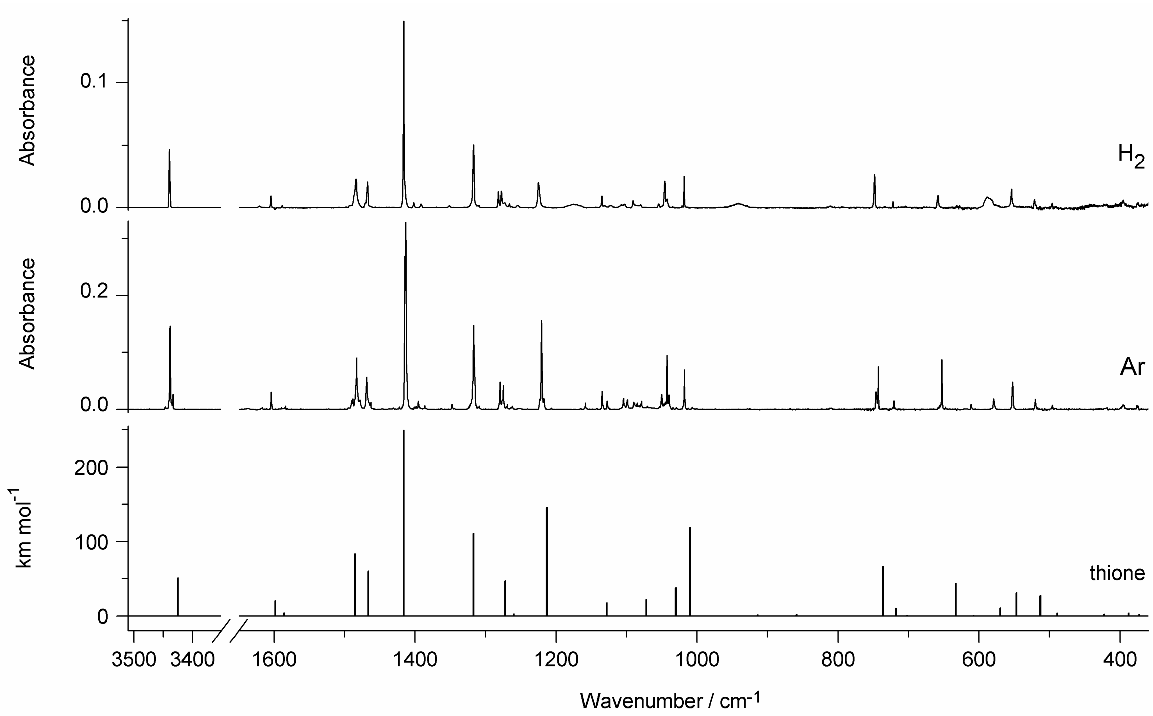

Figure S5. Fragments of the IR spectra of 2-thiobenzothiazole monomers isolated in: (upper panel) n-H_2 and (middle panel) Ar matrices; juxtaposed with (bottom panel) the theoretical spectrum calculated, at DFT(B3LYP)/6-311++G(2d,p) level, for the thione tautomer of the compound. The theoretical wavenumbers, calculated within the harmonic approximation, higher than 3000 cm^{-1} were multiplied by 0.95, whereas the wavenumbers lower than 3000 cm^{-1} were multiplied by 0.98.

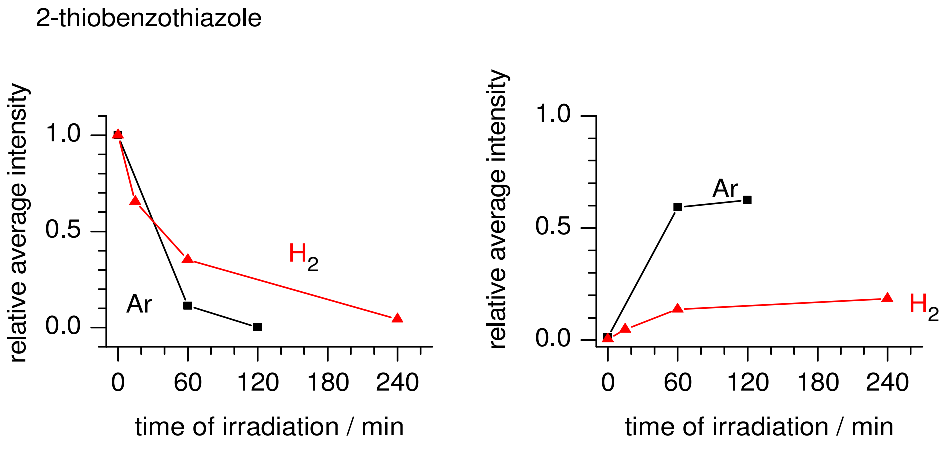

Decay of the thione tautomer during UV (305 nm) irradiation

Formation of the thiol tautomer during UV (305 nm) irradiation

Figure S6. The comparison of the progress of the thione → thiol phototautomeric reaction in monomers of 2-thiobenzothiazole isolated in Ar (black traces) and n-H_2 (red traces) matrices.

Table S2. Approximate assignment of the absorption bands, observed in the IR spectrum of monomers of 2-thiobenzothiazole isolated in Ar and n-H_2 matrices, to the normal modes calculated, at the DFT(B3LYP)/6-311++G(2d,p) level of theory, for the thione tautomer of the compound.

Experiment				Calculation		
Ar matrix		n-H_2 matrix		DFT(B3LYP)/ 6-311++G(2d,p)		Approximate description
$\tilde{\nu}$ cm^{-1}	*I* *rel.*	$\tilde{\nu}$ cm^{-1}	*I* *rel*	$\tilde{\nu}$ ^{a} cm^{-1}	A^{th} km mol^{-1}	
<u>3438</u>, 3433	99	3439	85	3425	51	ν NH
1604	12	1604	15	1598	20	ν CC, ν CN
1583	3	1588	3	1586	4	ν CC
1483	93	1484	111	1485	84	β NH, β CH
1469	56	1467	54	1466	60	β CH, ν CC
1414, <u>1413</u>	284	1416	275	1416	249	β NH, β CH
1317	137	1317	133	1317	111	ν CC, β CH
1280	29	1282	24	1272	47	β CH
1275	25	1277	32	1260	3	ν CN, β CH
1221	98	1225	80	1213	145	β NH, ν CN
<u>1135</u>, 1127	14	1135	17	1128	18	β CH
1104, 1099, 1089, 1085, 1079	36	1091, 1080	40	1072	22	β R, ν C-S
1050, 1043, 1040	80	1055, <u>1046</u>, 1043	72	1030	38	β CH
1018	30	1019	31	1010	119	ν C-S, ν C=S, β R
925	1	929	1	914	1	γ CH
868	2			859	2	β R, ν CN
746, 743	46	748	58	736	66	γ CH
721	7	722	6	718	10	γ CH, τ R
653	34	659	27	633	43	ν C-S, β R
612	4			608	1	β R, ν C-S
580	12			570	11	τ r, γ NH
553	28	554	47	547	31	γ NH, τ R
520	11	522	15	513	27	γ NH
496	3	496	7	489	4	β r, ν C-S
396	10	396	17	388	4	β r, ν C=S
376	4	376	6	373	2	β r, β R, β C=S

Table S2. *(continuation)*

[a] The theoretical wavenumbers higher than 3000 cm^{-1} were scaled by a factor of 0.95, whereas those lower than 3000 cm^{-1} were scaled by 0.98.
ν stretching, β bending, τ torsion, γ wagging,
β R bending of the six-membered ring, β r bending of the five-membered ring
The strongest bands are underlined; sh – shoulder

Table S3. Structures of the tautomeric forms of the studied compounds and the height of the barrier for the thione→thiol, oxo→hydroxy or N(1)H→N(7)H transformation, calculated at the DFT(B3LYP)/6-311++G(2d,p) level and at the MP2/6-311++G(2d,p) level.

Initial tautomer	⟶ Final tautomer	Barrier height / kJ mol^{-1}
3-thiopyridazine		
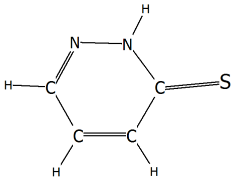	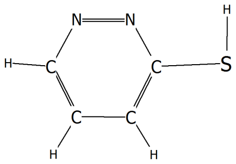	DFT 141<br>MP2 128
2-thioquinoline		
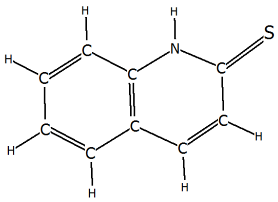	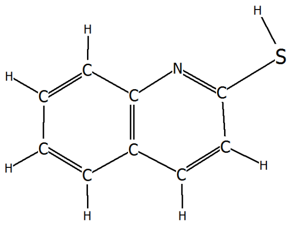	DFT 139<br>MP2 127
2-thioimidazole		
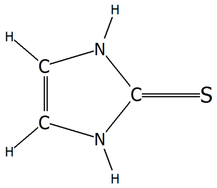	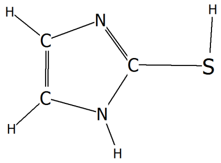	DFT 172<br>MP2 161
3-thio-1,2,4-triazole		
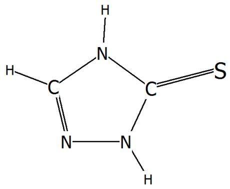	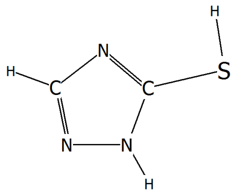	DFT 169<br>MP2 161
2-thiobenzothiazole		
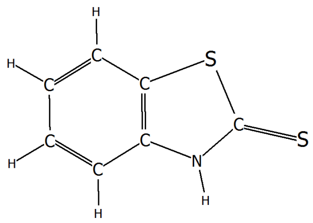	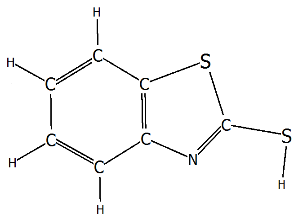	DFT 165<br>MP2 152

Compound	Structure 1	Structure 2	Values
thioacetamide	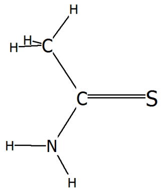	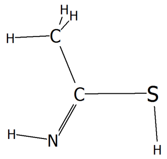	DFT 163<br>MP2 161
thiourea	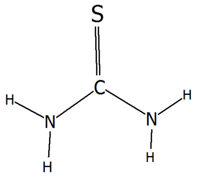	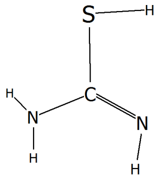	DFT 167<br>MP2 167
4-oxopyrimidine	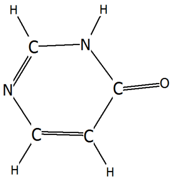	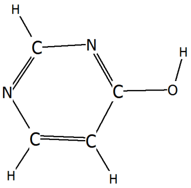	DFT 162<br>MP2 156
6-hydroxy-4-oxopyrimidine	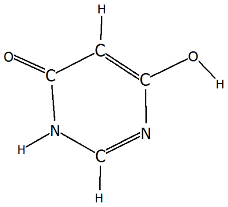	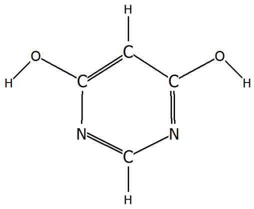	DFT 156<br>MP2 151
7-azaindole	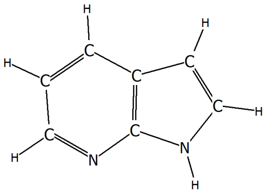	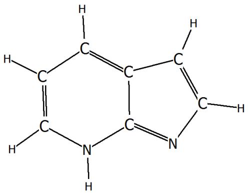	DFT 274<br>MP2 262
